# The Clinical Features of Inflammatory Bowel Disease in Patients with Obesity

**DOI:** 10.1155/2021/9981482

**Published:** 2021-08-02

**Authors:** Seong Kyun Kim, Ho-Su Lee, Beom-Jun Kim, Jin Hwa Park, Sung Wook Hwang, Dong-Hoon Yang, Byong Duk Ye, Jeong-Sik Byeon, Seung-Jae Myung, Suk-Kyun Yang, Sang Hyoung Park

**Affiliations:** ^1^Department of Gastroenterology, Asan Medical Center, University of Ulsan College of Medicine, Seoul, Republic of Korea; ^2^Department of Biochemistry, University of Ulsan College of Medicine, Asan Medical Center, Seoul, Republic of Korea; ^3^Department of Endocrinology and Metabolism, Asan Medical Center, University of Ulsan College of Medicine, Seoul, Republic of Korea

## Abstract

**Methods:**

We retrospectively reviewed the medical records of IBD patients who visited Asan Medical center. We used a large, well-characterized referral center-based cohort. The clinical features of IBD patients with body mass index (BMI) over 30 and matched controls with BMI under 30 were compared.

**Results:**

Among the 6,803 IBD patients enrolled in the Asan IBD Registry between June 1989 and December 2016, we identified 16 patients with Crohn's disease (CD) and 27 patients with ulcerative colitis (UC) whose BMI was over 30 at the time of diagnosis. Their clinical characteristics and course were compared with those of 64 and 108 matched patients with CD and UC, respectively. There were no significant differences in the risk of using steroids (hazards ratio (HR) = 0.633 and *P*=0.254), immunomodulators (HR = 0.831 and *P*=0.517), and anti-tumor necrosis factor (TNF) therapy (HR = 1.539 and *P*=0.351) and risk of bowel resections (HR = 1.858 and *P*=0.231) between CD patients with BMI over 30 and those with BMI under 30; similarly, UC patients did not show significant differences in the risk of using steroids (HR = 0.613 and *P*=0.145), immunomodulators (HR = 0.492 and *P*=0.111), anti-TNF therapy (HR = 0.385 and *P*=0.095), and risk of colectomy (HR = 0.262 and *P*=0.104). In the subgroup analysis, under-weight UC patients had a higher cumulative probability of needing steroids (HR = 0.2510 and *P*=0.042), needing immunomodulators (HR = 0.097 and *P*=0.014), and a higher risk of receiving colectomy (HR = 0.024 and *P*=0.019) than obese UC patients.

**Conclusions:**

Obese IBD patients with CD or UC did not show significantly different clinical features from nonobese IBD patients.

## 1. Introduction

Inflammatory bowel disease (IBD) includes a group of diseases featuring recurring and persistent chronic inflammation of the intestine, such as Crohn's disease (CD) and ulcerative colitis (UC). These are chronic, debilitating disorders that require lifelong medical treatment and even surgery [1, 2]. The pathogenesis of IBD is not fully understood but is thought to be multifactorial, involving variations in the patient's genome and environmental factors and alterations in the intestinal microbiota and mucosal immune response [3].

Obesity has become an important global public health issue in the current era. According to the World Health Organization's data, the prevalence of over-weight or obesity (body mass index (BMI) > 25 kg/m^2^) is as high as 35% in the global population, and it is increasing with time [4]. Globally, the cost of treating obesity and its related health complications may be as high as $2 trillion (US) [5]. The prevalence of IBD is also rising worldwide. Although IBD is usually associated with malnutrition and cachexia, the increasing incidence rates of IBD and obesity worldwide have led to the hypothesis that obesity plays a role in the occurrence of IBD. Understanding the interaction between obesity and IBD, in terms of disease pathogenesis, phenotypic disease expression, and response to therapy, is crucial to disease management.

Very few studies have investigated the clinical relevance of obesity and IBD, and most studies have focused on CD. In some studies, obesity was related to frequent perianal complications of CD and high rates of relapses and hospitalization [6] as well as short time to loss of response to anti-tumor necrosis factor (TNF) agents [7]. Other studies showed contradictory results and that obesity was associated with a good prognosis of IBD, including reduced risks of anti-TNF treatment, surgery, and hospitalization [8]. One study demonstrated negative prognostic implications, specifically regarding the risk of future hospitalization and corticosteroid use [9]. As such, the association between obesity and the features and course of IBD is controversial. Especially, there are very few studies on the clinical characteristics and prognosis of IBD in patients with obesity in the Korean population. In this study, we aimed to describe the clinical course and prognosis in Korean IBD patients with obesity and compare this group with a matched cohort.

## 2. Methods

### 2.1. Study Design and Population

The medical records of patients enrolled in the Asan IBD registry between June 1989 and December 2016 were retrospectively reviewed. The Asan IBD registry is a well-characterized referral center-based cohort of Koreans with IBD [10, 11] and includes details of IBD patients diagnosed and treated at Asan Medical Center. The diagnostic criteria of CD and UC in this study were based on the conventional clinical, radiologic, endoscopic, and histopathologic criteria, as described previously [12–14]. Patients aged below 17 years and those who were diagnosed with IBD before 1998 were excluded from this study.

To perform a matched case–control study, we defined cases and controls as follows: cases were defined as IBD patients with BMI over 30 at diagnosis and controls were patients with BMI under 30 at diagnosis. According to the Korean Society for the Study of Obesity, a BMI of 30 is classified as stage 2 obesity. The reasons for selecting a cutoff of stage 2 obesity were to avoid the inclusion of a large number of patients with stage 1 obesity and obtain clear results. The Asan IBD registry consisted of 6,803 IBD patients, among whom 6,127 patients had BMI data. We then isolated 311 patients whose BMI was over 30 of whom only 43 patients had their BMI measured within 6 months of diagnosis. The controls were matched to cases at a ratio of 4:1 for disease (type of IBD), sex, calendar year of diagnosis of IBD (±2 years), and age at diagnosis of IBD (±2.5 years). The study protocol was approved by the Institutional Review Board of the Asan Medical Center.

Data obtained from medical records were analyzed retrospectively. For comparing the clinical features of the disease between the groups, the sex, age, year at diagnosis of IBD, smoking status, and extent of IBD at diagnosis were retrieved. Then, we compared the clinical features of IBD, by calculating the cumulative probabilities of the use of medications (steroids, immunomodulators, and anti-TNF agents) and bowel resection.

Next, we selected two subgroups from the control group: under-weight patients with a BMI under 18.5 kg/m^2^ and normal-weight patients with a BMI from 18.5 to 25 kg/m^2^. We then compared the baseline characteristics and cumulative probabilities of medication use and bowel resection of each subgroup against those of the obese group (BMI over 30 kg/m^2^).

### 2.2. Statistical Analysis

Continuous variables were expressed as medians with ranges. Discrete data were tabulated as numbers and percentages. Categorical data were compared between cases and controls using the chi-squared test or Fisher's exact test, as appropriate. Continuous variables were compared using the Student's *t*-test or the Mann–Whitney *U* test. Cumulative probabilities of the use of medications and surgery were calculated using the Kaplan–Meier method. Comparison between cases and matched control patients was performed using the Cox proportional hazards regression. Statistical analyses were performed using SPSS (version 25.0; SPSS Inc., Chicago, IL). *P*-value <0.05 was considered statistically significant.

### 2.3. Ethical Considerations

The Institutional Review Board of Asan Medical Center approved the study protocol (IRB No. 2018-1193). The need to obtain written informed consent from each patient enrolled in the study was waived owing to the retrospective nature of this study.

## 3. Results

### 3.1. Baseline Characteristics of Patients

Among the 6,803 IBD patients (3,171 with CD and 3,632 with UC), 16 patients with CD and 27 patients with UC had BMI over 30 at the time of diagnosis. The clinical characteristics of the IBD patients according to the Montreal classification are shown in Tables 1 and 2.

Of the 16 obese CD patients, 11 (68.8%) were male, and the median age at diagnosis of CD was 21.3 years (range, 17.9–34.8 years). The median interval from onset to diagnosis was 9.4 months (range, 0–117.7 months), and the median follow-up after diagnosis of IBD was 102.1 months. All obese CD patients (100%) were treated with corticosteroids, 7 (43.8%) with immunomodulators, and 8 (50%) with anti-TNF agents. Seven patients (43.8%) underwent bowel resection. Among the UC patients, 21 (77.8%) were male, and the median age at diagnosis was 24 years (range, 16–69 years). The median interval from onset to diagnosis was 2.1 months (range, 0–24.7 months), and the median follow-up after diagnosis of IBD was 71.0 months. A total of 8 (29.6%) patients were treated with corticosteroids, 3 (11.1%) with immunomodulators, and 1 (3.7%) with anti-TNF agents. None of the patients underwent bowel resection.

The clinical characteristics and courses of 16 obese patients with CD and 27 obese patients with UC compared with those of 64 matched patients with CD and 108 matched patients with UC, respectively. The median BMI values at the time of CD diagnosis for patients with BMI over 30 and under 30 were 31.8 and 19.1, respectively, and the values at the time of UC diagnosis were 31.3 and 22.9, respectively. There were no significant differences between the obese and nonobese groups with respect to age at diagnosis, sex, smoking status, disease location, and disease course.

For more accurate comparisons, we selected two subgroups from the control group: under-weight patients with a BMI under 18.5 and normal-weight patients with a BMI from 18.5 to 25. There were 26 and 9 under-weight patients with CD and UC, respectively. There were 32 and 73 normal-weight patients with CD and UC, respectively. We then compared the baseline characteristics of each subgroup with those of the obese group. The results are shown in the supplementary materials (Supplementary Tables 1–4).

### 3.2. Clinical Outcomes

**Figure 1:**
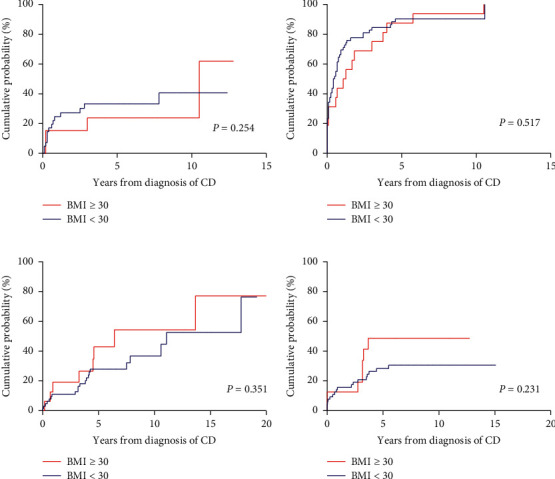
Cumulative probabilities of medication use and bowel resection in Crohn's disease patients with obesity and matched controls: (a) corticosteroids, (b) thiopurines, (c) anti-tumor necrosis factor (anti-TNF) agents, and (d) bowel resection.

**Figure 2:**
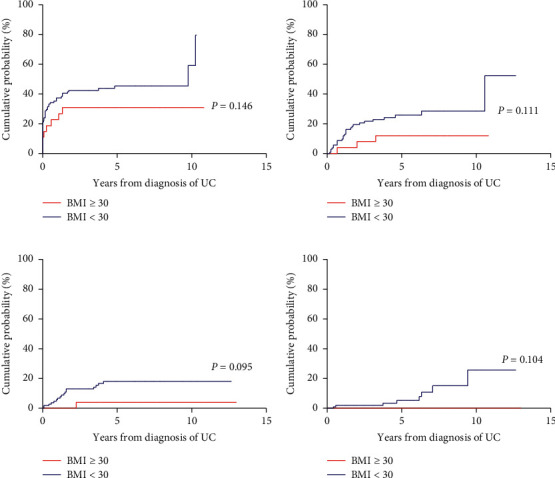
Cumulative probabilities of medication use and colectomy in ulcerative colitis patients with obesity and matched controls: (a) corticosteroids, (b) thiopurines, (c) anti-TNF agents, and (d) colectomy.

The cumulative probabilities of the need for steroids, immunomodulators, anti-TNF agents, and bowel resection of the CD and UC patients are presented in Figures 1 and 2. In the CD group, there were no significant differences in the risk of using steroids (hazards ratio (HR) = 0.633, 95% confidence interval (CI) = 0.289–1.388, and *P*=0.254), immunomodulators (HR = 0.831, 95% CI = 2.226–3.374, and *P*=0.517), and anti-TNF therapy (HR = 1.539, 95% CI = 0.136–1.022, and *P*=0.351), and risk of bowel resection (HR = 1.858, 95% CI = 0.674–5.119, and *P*=0.231) between patients with BMI over 30 and those with BMI under 30; similarly, in the UC group, there were no significant differences in the risk of using steroids (HR = 0.613, 95% CI = 0.317–1.185, and *P*=0.145), immunomodulators (HR = 0.492, 95% CI = 0.205–1.177, and *P*=0.111), and anti-TNF therapy (HR = 0.385, 95% CI = 0.125–1.180, and *P*=0.095) and risk of colectomy (HR = 0.262, 95% CI = 0.052–1.319, and *P*=0.104).

**Figure 3:**
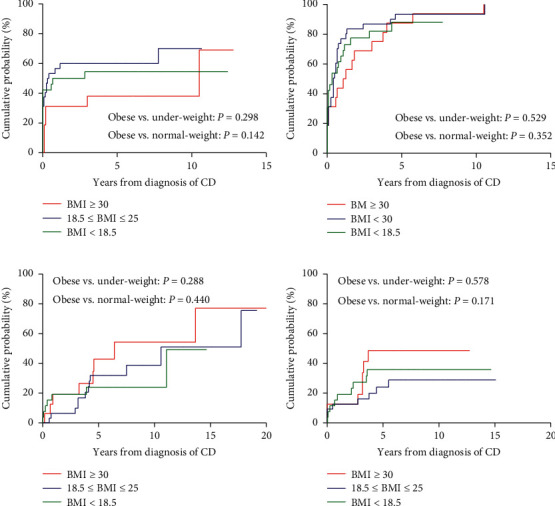
Cumulative probabilities of medication use and bowel resection in Crohn's disease patients with obesity and matched controls (subgroup analysis): (a) corticosteroids, (b) thiopurines, (c) anti-TNF agents, and (d) bowel resection.

**Figure 4:**
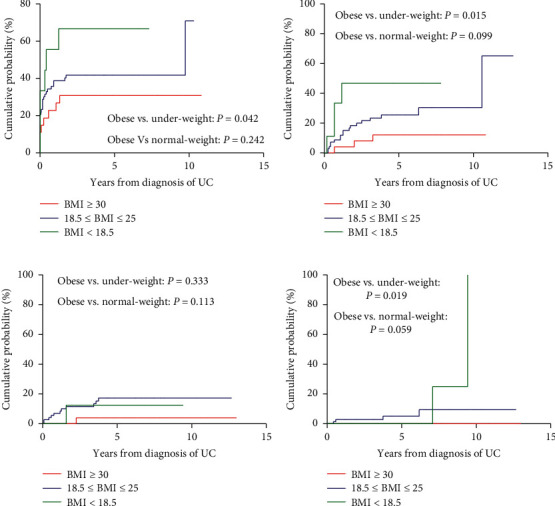
Cumulative probabilities of medication use and colectomy in ulcerative colitis patients with obesity and matched controls (subgroup analysis): (a) corticosteroids, (b) thiopurines, (c) anti-TNF agents, and (d) colectomy.

Additionally, we analyzed each subgroup's cumulative probabilities of the need for steroids, immunomodulators, anti-TNF agents, and bowel resection (Figures 3 and 4). Under-weight UC patients had a higher cumulative probability of needing steroids (HR = 0.2510; 95% confidence interval (CI) = 0.066–0.948, and *P*=0.042), immunomodulators (HR = 0.097, 95% CI = 0.015–0.629, and *P*=0.014), and a higher risk of receiving bowel resection (HR = 0.024, 95% CI = 0.001–0.545, and *P*=0.019) than obese UC patients.

## 4. Discussion

To the best of our knowledge, this is the first study to compare the clinical features of IBD in obese and nonobese patients in South Korea by calculating the cumulative probabilities of receiving different treatments.

The mechanisms by which obesity contributes to IBD can be largely explained in two ways. First, the large amount of adipose tissue may contribute to gut inflammation. Mesenteric visceral adipose tissue contains proinflammatory macrophages that secrete several inflammatory cytokines [15]. These inflammatory cytokines are thought to promote inflammatory responses in the gut [16]. Although overall obesity has not always been associated with severe disease activity and complications, visceral fat is thought to be independently associated with an increased risk of IBD-related complications [17, 18]. In one cross-sectional study, CD patients with complications had larger visceral fat areas on CT findings and higher visceral-to-subcutaneous fat area ratios than those without complications [19]. Second, intestinal dysbiosis may play a role in the development of IBD [20]. According to recent research, obesity induces intestinal barrier dysfunction that plays an important role in the pathogenesis of various obesity-induced diseases. Many studies have shown that obese patients have impaired intestinal barrier function, leading to enhanced translocation of bacteria or toxic bacterial products from the gut into the bloodstream and distant organs, resulting in systemic inflammation, insulin resistance, and tissue dysfunction [21–23]. This barrier dysfunction is a key pathogenic factor in IBD and obesity [24]. Hence, obesity may have an impact on the clinical outcome of IBD.

The results of previous studies on the association between obesity and the clinical outcomes of IBD vary, but we predicted that obesity would have a negative impact on the clinical outcomes and prognosis of IBD. In our study, there were no significant differences between the two groups. Perhaps, it would have been better to match over-weight with under-weight or over-weight with normal-weight patients. However, we compared the patients with BMI over 30 and those with BMI under 30, resulting in under-weight patients being grouped together with normal-weight patients for the analysis. This strategy may have obscured the association between obese and normal-weight patients by including sick patients in the comparator group, as low BMI could be a reflection of IBD activity [25]. Furthermore, low BMI may be the result rather than the cause of IBD activity, and obesity may be a reflection of a less aggressive or less severe form of IBD. This is supported by the findings of a recent study from Ireland, where obese or over-weight patients with CD had an overall less aggressive disease course than nonobese patients [26]. Third, the association between obesity and IBD is attracting attention as the global prevalence of obesity and IBD is increasing. However, the increase in the prevalence of IBD in Western countries has not been as drastic as the increase in the prevalence of obesity. Hence, the increase in the frequency of obesity in IBD patients merely reflects the rising frequency of obesity in the general population and that obesity is not contributing to the pathogenesis of IBD. To support this, a recent European epidemiologic study found no significant association between obesity and the development of IBD [27].

Our study has several limitations. As mentioned previously, under- and normal-weight patients were both included in the matched groups. To overcome this limitation, we selected under- and normal-weight subgroups from the control group and performed subgroup analysis. Under-weight UC patients had a higher cumulative probability of needing steroids and immunomodulators. Additionally, they had a higher risk of receiving bowel resection than obese UC patients. The rest of the analyses revealed no significant differences among the groups. The second limitation is the retrospective nature of the study, meaning selection bias could not be eliminated. A large proportion of patients were excluded because of missing data on BMI. Thus, we were only able to include 16 patients with CD and 27 patients with UC whose BMI was over 30 at the time of diagnosis. Further, a large proportion of patients with existing BMI data had a history of hospitalization, while many patients without a history of hospitalization did not have BMI data, and they were excluded from the study. Therefore, our study might have enrolled a larger proportion of IBD patients with worse clinical outcomes than the general IBD population, and this could have biased the results of our study. Third, the study cohort included patients only from a single tertiary referral center, which may have caused referral bias. Owing to the study's small sample size, the results need to be interpreted cautiously. We performed 1:4 case–control matching analysis to improve the statistical power of the study. Fourth, BMI is not a perfect measure of the degree of obesity, as theoretically, the effect of obesity on the body reflects the effect of adipose tissue on the body. Other measures that can determine the degree of obesity (adipose tissue) include waist circumference, waist–hip ratio, and visceral fat by cross-sectional imaging [28, 29]. Future studies should explore the relationship of BMI with visceral fat as measured by anthropometric measurements or imaging or the impact of visceral fat alone in patients with CD and UC. Finally, this study may have been improved if both groups contained the same proportion of smokers since smoking is an important driver of disease severity. However, we do not believe that the results of this study were confounded by smoking status because there were no significant differences between the obese and nonobese groups with respect to this variable.

In conclusion, there were no significant differences in clinical features represented by cumulative probabilities of receiving different treatments between the obese and the nonobese patients with IBD. Further prospective, long-term follow-up studies are needed to confirm these observations.

## Figures and Tables

**Table 1:** Demographic and clinical characteristics of patients with obesity and matched controls: Crohn's disease.

	Crohn's disease (BMI > 30)	Crohn's disease (BMI < 30)	*P*-value
No. of patients	16	64	
Median BMI at diagnosis (range)	31.8 (30.1–41.5)	19.1 (14.5–29.3)	
Male (%)	11 (68.8)	44 (68.8)	1.000
Median age at diagnosis (range), year	21.3 (17.9–34.8)	21.2 (17.9–35.9)	0.957
Median interval from onset to diagnosis (range), months	9.4 (0.0–117.7)	9.5 (0.1–108.2)	0.995
Mean follow-up after diagnosis of IBD (months)	102.1 ± 58.4	91.0 ± 48.6	0.435
Smoking status at diagnosis (%)			0.517
Current smokers	5 (31.3)	20 (31.3)	
Ex-smokers	1 (6.3)	1 (1.6)	
Never-smokers	10 (62.5)	43 (67.2)	
Disease location at diagnosis (Montreal classification)			0.712
L1 (terminal ileum)	5 (31.3)	14 (21.9)	
L2 (colon)	1 (6.3)	4 (6.3)	
L3 (ileocolon)	10 (62.5)	46 (71.9)	
L4 (upper GI modifier)	4 (25.0)	16 (25.0)	1.000
Disease location, final (Montreal classification)			1.000
L1 (terminal ileum)	4 (25.0)	14 (21.9)	
L2 (colon)	0 (0.0)	3 (4.7)	
L3 (ileocolon)	12 (75.0)	47 (73.4)	
L4 (upper GI modifier)	4 (25.0)	18 (28.1)	1.000
Perianal fistula			0.592
At diagnosis	8 (50.0)	24 (37.5)	
Occurrence during follow-up	1 (6.3)	8 (12.5)	
Never	7 (43.8)	32 (50.0)	
Disease behavior at diagnosis (Montreal classification)			1.000
B1 (nonstricturing and nonpenetrating)	13 (81.3)	48 (75.0)	
B2 (stricturing)	1 (6.25)	5 (7.8)	
B3 (penetrating)	2 (12.5)	11 (17.2)	
Disease behavior, final (Montreal classification)			0.717
B1 (nonstricturing and nonpenetrating)	9 (56.3)	41 (64.1)	
B2 (stricturing)	2 (12.5)	10 (15.6)	
B3 (penetrating)	5 (31.3)	13 (20.3)	
Medication history			
Steroids	7 (43.8)	37 (57.8)	0.334
Immunomodulators	16 (100.0)	57 (89.1)	0.312
Anti-tumor necrosis factor therapy	8 (50.0)	21 (32.8)	0.201
Surgical outcomes			
Bowel resection	7 (43.8)	18 (28.1)	0.228

GI, gastrointestinal, IBD, inflammatory bowel disease, UC, ulcerative colitis, and BMI, body mass index.

**Table 2:** Demographic and clinical characteristics of patients with obesity and matched controls: ulcerative colitis.

	Ulcerative colitis (BMI > 30)	Ulcerative colitis (BMI < 30)	*P*-value
No. of patients	27	108	
Median BMI at diagnosis (range)	31.3 (30.3–37.1)	22.9 (14.0–29.3)	
Male (%)	21 (77.8)	84 (77.8)	1.000
Median age at diagnosis (range), years	42 (16–69)	42 (16–71)	0.974
Median interval from onset to diagnosis (range), months	2.1 (0–24.7)	2.9 (0–63.8)	0.483
Mean follow-up after diagnosis of IBD (months)	71.0 ± 34.6	62.1 ± 33.1	0.219
Smoking status at diagnosis (%)			0.648
Current smokers	7 (25.9)	23 (21.3)	
Ex-smokers	7 (25.9)	38 (35.2)	
Never-smokers	13 (48.1)	47 (43.5)	
UC extent (at diagnosis)			0.883
Proctitis	12 (44.4)	52 (48.1)	
Left-sided colitis	8 (29.6)	27 (25.0)	
Extensive colitis	7 (25.9)	29 (26.9)	
UC extent (worst ever)			0.467
Proctitis	10 (37.0)	42 (38.9)	
Left-sided colitis	10 (37.0)	28 (25.9)	
Extensive colitis	7 (25.9)	38 (35.2)	
Medication history			
Steroids	8 (29.6)	49 (45.4)	0.139
Immunomodulators	3 (11.1)	28 (25.9)	0.102
Anti-tumor necrosis factor therapy	1 (3.7)	17 (15.7)	0.083
Surgical outcomes			
Colectomy	0 (0.0)	8 (7.4)	0.159

GI, gastrointestinal, IBD, inflammatory bowel disease, UC, ulcerative colitis, and BMI, body mass index.

## Data Availability

The data underlying this article cannot be shared publicly due to the privacy of individuals that participated in the study.

## Supplementary Materials

Supplementary Materials Supplementary Table 1: demographic and clinical characteristics of patients with obesity and under-weight patients: Crohn's disease. Supplementary Table 2: demographic and clinical characteristics of patients with obesity and under-weight patients: ulcerative colitis. Supplementary Table 3: demographic and clinical characteristics of patients with obesity and normal-weight patients: Crohn's disease. Supplementary Table 4: demographic and clinical characteristics of patients with obesity and normal-weight patients: ulcerative colitis.
