# Supplementary material for: The Clinical Features of Inflammatory Bowel Disease in Patients with Obesity
Source: Can J Gastroenterol Hepatol. 2021 Aug 2;2021:9981482. doi: 10.1155/2021/9981482 (PMC8352714; doi:10.1155/2021/9981482)
Supplement: Supplementary Materials — Supplementary Table 1: demographic and clinical characteristics of patients with obesity and under-weight patients: Crohn's disease. Supplementary Table 2: demographic and clinical characteristics of patients with obesity and under-weight patients: ulcerative colitis. Supplementary Table 3: demographic and clinical characteristics of patients with obesity and normal-weight patients: Crohn's disease. Supplementary Table 4: demographic and clinical characteristics of patients with obesity and normal-weight patients: ulcerative colitis. [file 9981482.f1.docx]

| **Supplementary table 1.** Demographic and clinical characteristics of patients with obesity and underweight patients: Crohn’s disease | | | |
| --- | --- | --- | --- |
|  | **Crohn's disease (BMI>30** **kg/m^2^)** | **Crohn's disease (BMI<18.5 kg/m^2^)** | **P-value** |
| **No. of patients** | 16 | 26 |  |
| **Median BMI at diagnosis (range)** | 31.8 (30.1–41.5) | 17.0 (14.5–18.4) |  |
| **Male (%)** | 11 (68.8) | 17 (65.4) | 0.957 |
| **Median age at diagnosis (range), year** | 21.3 (17.9–34.8) | 20 (18–36) | 0.746 |
| **Median interval from onset to diagnosis (range), months** | 9.4 (0.0–117.7) | 12.1 (0.6–108.2) | 0.698 |
| **Mean follow-up after diagnosis of IBD (months)** | 88.5 (8.0–250.0) | 83.5 (18.0–176.0) | 0.193 |
| **Smoking status at diagnosis (%)** |  |  | 0.429 |
| Current smokers | 5 (31.3) | 8 (30.8) |  |
| Ex-smokers | 1 (6.3) | 0 (0.0) |  |
| Never-smokers | 10 (62.5) | 18 (69.2) |  |
| **Disease location at diagnosis (Montreal classification)** |  |  | 0.421 |
| L1 (Terminal ileum) | 5 (31.3) | 1 (3.8) |  |
| L2 (Colon) | 1 (6.3) | 1 (3.8) |  |
| L3 (Ileocolon) | 10 (62.5) | 24 (92.3) |  |
| L4 (Upper GI modifier) | 4 (25.0) | 3 (11.5) | 0.256 |
| **Disease location, final (Montreal classification)** |  |  | 0.566 |
| L1 (Terminal ileum) | 4 (25.0) | 4 (15.4) |  |
| L2 (Colon) | 0 (0.0) | 1 (3.8) |  |
| L3 (Ileocolon) | 12 (75.0) | 21 (80.8) |  |
| L4 (Upper GI modifier) | 4 (25.0) | 4 (15.4) | 0.441 |
| **Perianal fistula** |  |  | 0.849 |
| At diagnosis | 8 (50.0) | 12 (46.2) |  |
| Occurrence during follow-up | 1 (6.3) | 3 (11.5) |  |
| Never | 7 (43.8) | 11 (42.3) |  |
| **Disease behavior at diagnosis (Montreal classification)** |  |  | 0.669 |
| B1 (Nonstricturing, nonpenetrating) | 13 (81.3) | 18 (69.2) |  |
| B2 (Stricturing) | 1 (6.25) | 2 (7.7) |  |
| B3 (Penetrating) | 2 (12.5) | 6 (23.1) |  |
| **Disease behavior, final (Montreal classification)** |  |  | 0.994 |
| B1 (Nonstricturing, nonpenetrating) | 9 (56.3) | 15 (57.7) |  |
| B2 (Stricturing) | 2 (12.5) | 3 (11.5) |  |
| B3 (Penetrating) | 5 (31.3) | 8 (30.8) |  |
| **Medication history** |  |  |  |
| Steroids | 7 (43.8) | 14 (53.8) | 0.525 |
| Immunomodulators | 16 (100.0) | 22 (84.6) | 0.099 |
| Anti-tumor necrosis factor therapy | 8 (50.0) | 7 (26.9) | 0.130 |
| **Surgical outcomes** |  |  |  |
| Bowel resection | 7 (43.8) | 9 (34.6) | 0.554 |
| GI, gastrointestinal; IBD, inflammatory bowel disease; UC, ulcerative colitis; BMI, body mass index | | |  |

| **Supplementary table 2.** Demographic and clinical characteristics of patients with obesity and underweight patients: Ulcerative colitis | | | | |
| --- | --- | --- | --- | --- |
|  | | **Ulcerative colitis (BMI>30 kg/m^2^)** | **Ulcerative colitis (BMI<18.5 kg/m^2^)** | **P-value** |
| **No. of patients** | 27 | | 9 |  |
| **Median BMI at diagnosis (range)** | 31.3 (30.3–37.1) | | 17.2 (14.0–18.3) |  |
| **Male (%)** | 21 (77.8) | | 6 (66.7) | 0.505 |
| **Median age at diagnosis (range), years** | 42 (16–69) | | 36 (16–61) | 0.247 |
| **Median interval from onset to diagnosis (range), months** | 2.1 (0–24.7) | | 1.0 (0.1–3.0) | 0.073 |
| **Mean follow-up after diagnosis of IBD (months)** | 73 (2–156) | | 85.0 (9.0–121.0) | 0.843 |
| **Smoking status at diagnosis (%)** |  | |  | 0.301 |
| Current smokers | 7 (25.9) | | 1 (11.1) |  |
| Ex-smokers | 7 (25.9) | | 1 (11.1) |  |
| Never-smokers | 13 (48.1) | | 7 (77.8) |  |
| **UC extent (at diagnosis)** |  | |  | 0.834 |
| Proctitis | 12 (44.4) | | 3 (33.3) |  |
| Left-sided colitis | 8 (29.6) | | 3 (33.3) |  |
| Extensive colitis | 7 (25.9) | | 3 (33.3) |  |
| **UC extent (worst ever)** |  | |  | 0.541 |
| Proctitis | 10 (37.0) | | 3 (33.3) |  |
| Left-sided colitis | 10 (37.0) | | 2 (22.2) |  |
| Extensive colitis | 7 (25.9) | | 4 (44.4) |  |
| **Medication history** |  | |  |  |
| Steroids | 8 (29.6) | | 6 (66.7) | 0.048 |
| Immunomodulators | 3 (11.1) | | 4 (44.4) | 0.029 |
| Anti-tumor necrosis factor therapy | 1 (3.7) | | 1 (11.1) | 0.401 |
| **Surgical outcomes** |  | |  |  |
| Colectomy | 0 (0.0) | | 2 (22.2) | 0.012 |
| GI, gastrointestinal; IBD, inflammatory bowel disease; UC, ulcerative colitis; BMI, body mass index | | | |  |

| **Supplementary table 3.** Demographic and clinical characteristics of patients with obesity and normal weight patients: Crohn’s disease | | | |
| --- | --- | --- | --- |
|  | **Crohn's disease (BMI>30 kg/m^2^)** | **Crohn's disease (18.5 kg/m^2^≤BMI≤25 kg/m^2^)** | **P-value** |
| **No. of patients** | 16 | 32 |  |
| **Median BMI at diagnosis (range)** | 31.8 (30.1–41.5) | 20.1 (18.5–23.8) |  |
| **Male (%)** | 11 (68.8) | 22 (68.8) | 1.000 |
| **Median age at diagnosis (range), year** | 21.3 (17.9–34.8) | 22 (18–35) | 0.810 |
| **Median interval from onset to diagnosis (range), months** | 9.4 (0.0–117.7) | 6.4 (0.1–105.8) | 0.437 |
| **Mean follow-up after diagnosis of IBD (months)** | 88.5 (8.0–250.0) | 88.5 (4.0–230.0) | 0.973 |
| **Smoking status at diagnosis (%)** |  |  | 0.875 |
| Current smokers | 5 (31.3) | 10 (31.2) |  |
| Ex-smokers | 1 (6.3) | 1 (3.1) |  |
| Never-smokers | 10 (62.5) | 21 (65.6) |  |
| **Disease location at diagnosis (Montreal classification)** |  |  | 0.974 |
| L1 (Terminal ileum) | 5 (31.3) | 9 (28.1) |  |
| L2 (Colon) | 1 (6.3) | 1 (3.1) |  |
| L3 (Ileocolon) | 10 (62.5) | 22 (68.8) |  |
| L4 (Upper GI modifier) | 4 (25.0) | 13 (40.6) | 0.386 |
| **Disease location, final (Montreal classification)** |  |  | 0.742 |
| L1 (Terminal ileum) | 4 (25.0) | 9 (28.1) |  |
| L2 (Colon) | 0 (0.0) | 1 (3.1) |  |
| L3 (Ileocolon) | 12 (75.0) | 22 (68.8) |  |
| L4 (Upper GI modifier) | 4 (25.0) | 13 (40.6) | 0.286 |
| **Perianal fistula** |  |  | 0.128 |
| At diagnosis | 8 (50.0) | 7 (21.9) |  |
| Occurrence during follow-up | 1 (6.3) | 5 (15.6) |  |
| Never | 7 (43.8) | 20 (62.5) |  |
| **Disease behavior at diagnosis (Montreal classification)** |  |  | 0.882 |
| B1 (Nonstricturing, nonpenetrating) | 13 (81.3) | 24 (75.0) |  |
| B2 (Stricturing) | 1 (6.25) | 3 (9.4) |  |
| B3 (Penetrating) | 2 (12.5) | 5 (15.6) |  |
| **Disease behavior, final (Montreal classification)** |  |  | 0.403 |
| B1 (Nonstricturing, nonpenetrating) | 9 (56.3) | 20 (62.5) |  |
| B2 (Stricturing) | 2 (12.5) | 7 (21.9) |  |
| B3 (Penetrating) | 5 (31.3) | 5 (15.6) |  |
| **Medication history** |  |  |  |
| Steroids | 7 (43.8) | 20 (62.5) | 0.217 |
| Immunomodulators | 16 (100.0) | 30 (93.8) | 0.307 |
| Anti-tumor necrosis factor therapy | 8 (50.0) | 12 (37.5) | 0.408 |
| **Surgical outcomes** |  |  |  |
| Bowel resection | 7 (43.8) | 8 (25.0) | 0.186 |
| GI, gastrointestinal; IBD, inflammatory bowel disease; UC, ulcerative colitis; BMI, body mass index | | |  |

| **Supplementary table 4.** Demographic and clinical characteristics of patients with obesity and normal weight patients: Ulcerative colitis | | | | |
| --- | --- | --- | --- | --- |
|  | | **Ulcerative colitis (BMI>30 kg/m^2^)** | **Ulcerative colitis (18.5 kg/m^2^≤BMI≤25 kg/m^2^)** | **P-value** |
| **No. of patients** | 27 | | 73 |  |
| **Median BMI at diagnosis (range)** | 31.3 (30.3–37.1) | | 22.2 (18.5–24.9) |  |
| **Male (%)** | 21 (77.8) | | 53 (72.6) | 0.708 |
| **Median age at diagnosis (range), years** | 42 (16–69) | | 42 (16–71) | 0.227 |
| **Median interval from onset to diagnosis (range), months** | 2.1 (0–24.7) | | 3.3 (0–63.8) | 0.018 |
| **Mean follow-up after diagnosis of IBD (months)** | 73 (2–156) | | 58.0 (0.7–152.0) | 0.018 |
| **Smoking status at diagnosis (%)** |  | |  | 0.205 |
| Current smokers | 7 (25.9) | | 15 (20.5) |  |
| Ex-smokers | 7 (25.9) | | 24 (32.9) |  |
| Never-smokers | 13 (48.1) | | 34 (46.6) |  |
| **UC extent (at diagnosis)** |  | |  | 0.751 |
| Proctitis | 12 (44.4) | | 34 (46.6) |  |
| Left-sided colitis | 8 (29.6) | | 19 (26.0) |  |
| Extensive colitis | 7 (25.9) | | 20 (27.4) |  |
| **UC extent (worst ever)** |  | |  | 0.872 |
| Proctitis | 10 (37.0) | | 29 (39.7) |  |
| Left-sided colitis | 10 (37.0) | | 18 (24.7) |  |
| Extensive colitis | 7 (25.9) | | 26 (35.6) |  |
| **Medication history** |  | |  |  |
| Steroids | 8 (29.6) | | 31 (42.5) | 0.169 |
| Immunomodulators | 3 (11.1) | | 20 (27.4) | 0.289 |
| Anti-tumor necrosis factor therapy | 1 (3.7) | | 11 (15.1) | 0.751 |
| **Surgical outcomes** |  | |  |  |
| Colectomy | 0 (0.0) | | 4 (5.5) | 0.069 |
| GI, gastrointestinal; IBD, inflammatory bowel disease; UC, ulcerative colitis; BMI, body mass index | | | |  |
